# Individual aggregates of amyloid beta induce temporary calcium influx through the cell membrane of neuronal cells

**DOI:** 10.1038/srep31910

**Published:** 2016-08-24

**Authors:** Anna Drews, Jennie Flint, Nadia Shivji, Peter Jönsson, David Wirthensohn, Erwin De Genst, Cécile Vincke, Serge Muyldermans, Chris Dobson, David Klenerman

**Affiliations:** 1 Department of Chemistry, University of Cambridge, Lensfield Road, Cambridge, UK; 2 Department of Chemistry, Lund University, SE-22100 Lund, Sweden; 3 Laboratory of Cellular and Molecular Immunology, Vrije Universiteit Brussel, Brussel, Belgium

## Abstract

Local delivery of amyloid beta oligomers from the tip of a nanopipette, controlled over the cell surface, has been used to deliver physiological picomolar oligomer concentrations to primary astrocytes or neurons. Calcium influx was observed when as few as 2000 oligomers were delivered to the cell surface. When the dosing of oligomers was stopped the intracellular calcium returned to basal levels or below. Calcium influx was prevented by the presence in the pipette of the extracellular chaperone clusterin, which is known to selectively bind oligomers, and by the presence a specific nanobody to amyloid beta. These data are consistent with individual oligomers larger than trimers inducing calcium entry as they cross the cell membrane, a result supported by imaging experiments in bilayers, and suggest that the initial molecular event that leads to neuronal damage does not involve any cellular receptors, in contrast to work performed at much higher oligomer concentrations.

A pathological hallmark of Alzheimer’s disease (AD) is the presence of extracellular plaques composed of amyloid beta fibrils in the hippocampus and neocortex of the brain^1^,^2^,^3^. Amyloid beta (Aβ) is formed by proteolytic processing of the transmembrane amyloid precursor protein by beta and gamma secretase. It aggregates to form small oligomers which then self-assemble into protofibrils and fibrils which are deposited as plaques. There is significant evidence that the plaques themselves are not toxic; indeed, it appears that the true agents of toxicity are the small soluble oligomers^4^,^5^,^6^,^7^. Although Aβ has been implicated in Alzheimer’s disease since the early 1980s, the primary target for Aβ oligomers and the mechanism of their toxicity remain elusive and include specific binding to a range of cellular receptors as well as disruption to the cell membrane and formation of pores in the cell membrane^8^,^9^. This important question has not been addressed to date due to a number of factors. Firstly, there has been a lack of methods to reproducibly make and characterise Aβ oligomers and secondly, the experiments to probe interactions of these oligomers with cells are often performed at oligomer and monomer concentrations much higher than those that occur under physiological conditions. In addition many cellular responses in these experiments are observed in minutes or hours, including cell death, raising questions of why it takes decades to develop the disease.

Experiments have been previously performed directly using human cerebral spinal fluid (CSF) from Alzheimer’s patients, without any preparation steps. This has shown that the Aβ oligomers present can induce long-term potentiation deficit in brain slices which can be prevented by the addition of antibodies to Aβ^10^. CSF from Alzheimer’s patients has also been shown to cause cell toxicity which can be prevented by addition of physiological amounts of extracellular chaperones^11^, such as clusterin. Furthermore, more recently a sensitive ELISA based method has been developed to directly measure the Aβ oligomer concentration in CSF and used to show that this is approximately 0.5 pM in patients with Alzheimer’s disease^12^. Taken together these results suggest that low pM concentrations of Aβ oligomers are capable of inducing neuronal damage but there have been no reported studies of the damage mechanism at these low concentrations.

We have also previously studied the effect of synthetic oligomers of Aβ_40_ and Aβ_42_ on primary neuronal cells, as a function of oligomer dose^13^. In this study we used fluorophore labelled peptide so that single molecule fluorescence detection could be used to characterise the concentration and relative size of the oligomers used in these experiments. The oligomers ranged in size from dimers to 30mers, decaying exponentially with oligomer size, so that most of the oligomers were small oligomers less than 10mers. Our results show that it is possible to observe calcium oscillations in astrocytes, but not neurons, at oligomer concentrations down to 200 pM, a concentration 100 fold higher concentration than the oligomer concentration in human CSF^12^. The calcium oscillations, which were due to extracellular calcium entering the cell, led to reactive oxygen species (ROS) production and then caspase 3 activation in both astrocytes and neurons. These data are consistent with previous studies that show that the first cell-type affected by Aβ oligomers are astrocytes^14^,^15^.

In this work we have used a nanopipette to locally deliver Aβ oligomers to astrocytes to control the location and number of oligomers applied to an individual cell in order to gain more detailed insights into the molecular basis of the oligomer induced calcium influx, and how it depends on the number of oligomers that the cell encounters. A schematic of the experiment is shown in Fig. 1. Our method is based on Scanning Ion Conductance Microscopy (SICM)^16^, where a change in pipette current provides a real-time feedback to allow a nanopipette to maintain a controlled distance over a cell^17^, and can easily be combined with fluorescence imaging. We have used the nanopipette for controlled voltage and pressure driven delivery of small molecules, proteins or antibodies to defined positions on a surface^18^.

We have previously measured and modelled the delivery of reagents from the nanopipette as a function of pipette size, distance from the surface and the applied voltage and pressure^19^. This model is used to estimate the number of Aβ oligomers delivered from the nanopipette. In this work only a region of about 1 μm^2^ is dosed with oligomers from a pipette held 300 nm above the cell surface, so there is only a small distance that molecules need to diffuse across before encountering the cell surface. This significantly increases the local flux of oligomers reaching the cell surface compared to bath application, due to an oligomer depletion layer outside the cell surface in the latter case. The use of SICM, therefore, allows us to have a constant and defined dose of oligomers to the cell surface. This enables us to perform highly quantitative measurement of the effect of low concentrations of oligomers on calcium influx on neuronal cells.

## Results

### Characterisation of synthetic oligomers of Aβ_42_

We have previously shown that oligomers made using fluorophore labelled Aβ peptide are as cytotoxic to cells as the unlabelled peptide, while the presence of the fluorophores allows us to measure the concentration of oligomers present using single molecule fluorescence^20^. We firstly characterised the synthetic oligomers of Aβ_42_ formed during an aggregation reaction using Alexa Fluor 488 and HiLyte Fluor 647 labelled Aβ_42_ at 500 nM monomer. In these experiments we directly count the number of oligomers present since they give rise to coincident bursts of fluorescence. We can also use the brightness of the coincident burst from the oligomers to estimate the oligomer size distribution. Aggregation for 5 hours, with shaking, resulted in the formation of approximately 3 nM ± 0.5 nM oligomers, with a size distribution from dimers to 20mers, as measured by single molecule confocal microscopy. We observed no change in oligomer size distribution with time during the aggregation. A representative size histogram, obtained using single molecule confocal fluorescence measurements, is shown in Fig. 2 after 6 hours aggregation.

We also used a new aggregate-specific method to characterise the Aβ oligomers. This method detects surface-adsorbed aggregates by use of the dye Thioflavin T (ThT), which fluoresces when bound to oligomers, combined with an ultra-sensitive total internal reflection fluorescence microscopy (TIRFM)^21^. The advantage of this approach was that monomers gave no signal. This method allowed us to count the number of oligomers present and measure their relative brightness. Experiments were performed with HiLyte Fluor 647 Aβ_42_ only, since the Alexa Fluor 488 fluorescence overlaps with the ThT fluorescence, and these samples were imaged using ThT after dilution by a factor of 200 corresponding to an oligomer concentration of 15 pM. The results are shown in Fig. 2B. This provided a simpler method to ensure that Aβ oligomer preparations used in the dosing experiments were reproducible.

### Astrocyte dosing experiments

**Figure 1:**
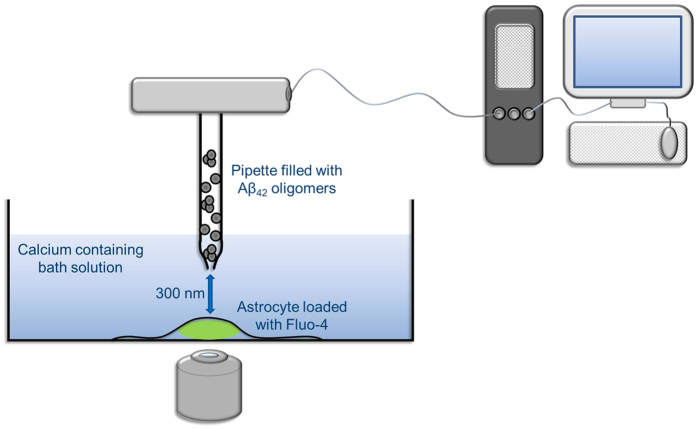
Schematic of the SICM experiment to measure the calcium influx in astrocytes.

**Figure 2:**
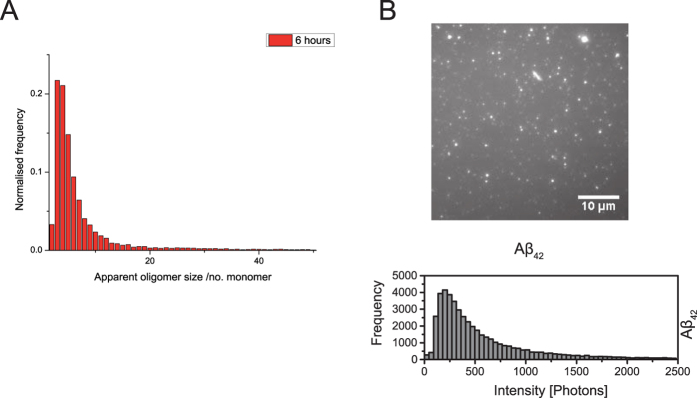
Characterisation of synthetic oligomers of Aβ_42_. (**A**) Oligomer size distribution after 6 hours aggregation measured using single molecule confocal microscopy. (**B**) Total internal reflection fluorescence microscopy image of oligomers on a glass surface, using Thioflavin T, and the corresponding brightness histogram of 27 field of views.

**Figure 3:**
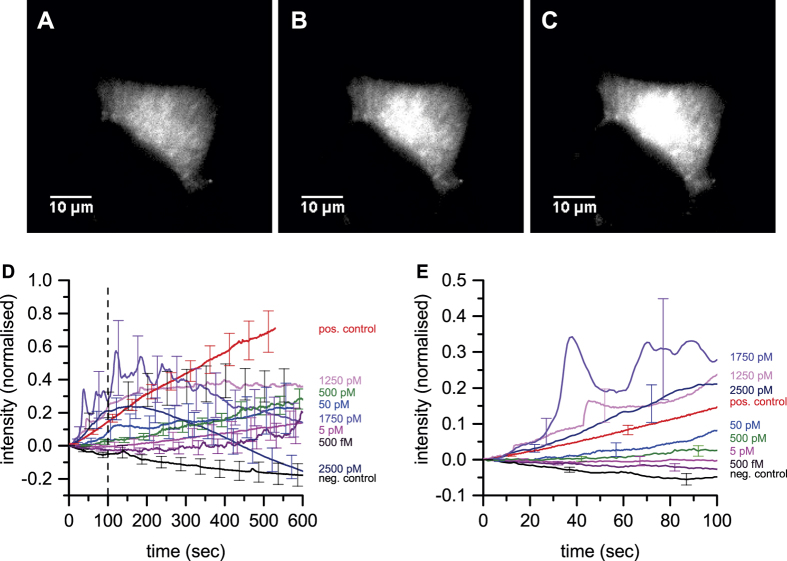
Effect of dosing synthetic Aβ_42_ oligomers on intracellular calcium of astrocytes. (**A–C**) Representative data showing an astrocyte treated with an average surface concentration of 500 pM Aβ_42_ oligomers. (**A**) At the start of the experiment, (**B**) after 5 min, (**C**) after 10 min. (**D**) The average change in the normalised intracellular calcium of astrocytes with Aβ_42_ oligomer surface concentrations from 500 fM−25 nM applied. Movies were taken over 10 min. Images were taken every 400 msec to 1 sec (n is greater than 6 for each trace). For the negative control L15 buffer only was dosed from the nanopipette and for the positive control 2 μM Ionomycin was applied. The error bars, SEM, are shown every 50 seconds. The n value for each trace is tabulated in supplementary materials.

We initially performed experiments on astrocytes since we had previously observed that oligomers induced calcium influx in astrocytes using bath application, and shown that astrocytes are the first cells that the oligomers damage^20^. Astrocytes from a mixed glial preparation were loaded with the calcium sensitive dye Fluo-4 and kept in Leibovitz’s 15 medium (L15) to investigate the calcium influx into the cells due to the application of Aβ_42_ oligomers (Fig. 1). Solutions containing samples of different concentrations of oligomers and monomer, were locally delivered, via the SICM nanopipette, to the surface of individual astrocytes by application of a combination of pressure and voltage. In these experiments the pipette is controlled 300 nm above the cell surface and locally applies oligomers within an area of about 1 μm^2^. Previous work has shown that the opening of pores in the cell membrane has no effect on the performance of the SICM^22^. We have developed a quantitative model for dosing from the nanopipette (see Supplementary materials). The calcium influx that resulted from oligomer dosing was recorded via an EMCCD camera (Fig. 3A–C and Supplementary Movie 1). Initial experiments showed that frequent Ca^2+^ oscillations were observed in addition to a steady rise in intracellular calcium on application of Aβ_42_ oligomers. 6-Methyl-2-(phenylethynyl)pyridine hydrochloride (MPEP), a specific metabotropic glutamate receptor 5 (mGlu5) blocker, was added to the bath to prevent these Ca^2+^ oscillations (see Supplementary materials). Control experiments showed there was no increase in intracellular calcium on addition of L15 medium alone from the pipette or just synthetic monomer (Supplementary Figures S1 and S2). Since the uptake of Fluo-4 varies between cells we normalised the calcium traces to the initial level of calcium at the start of the experiment, so we could directly compare the results from different cells.

**Figure 4:**
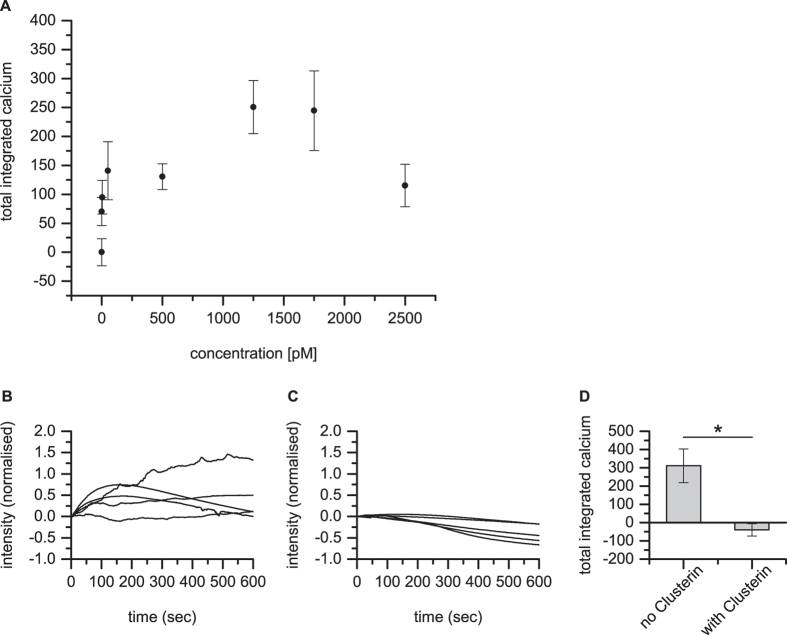
Aβ_42_ oligomer dose dependency and the prevention of oligomer-induced calcium influx by clusterin. (**A**) Analysis of data in Fig. 3. Total integrated calcium entering an astrocyte as a function of oligomer concentration at the cell surface over the 10 minute experiment. There was a small reduction in integrated calcium in control cells, due to loss of dye and photobleaching over the 10 minute experiment. This was measured in control experiments and has been subtracted from all the data. Error bars are SEM. (**B,C**) Normalised calcium traces for an astrocyte with 500 pM oligomer concentration at the cell surface over the 10 minute experiment in the presence (n = 5), (**B**) and absence, (**C**) of 2 μg/mL clusterin in the pipette (n = 5). The oligomers were incubated with clusterin for 15 minutes before dosing was started. (**D**) Total integrated calcium for the data in (**B**,**C**) corrected for photobleaching and dye loss. The p value was 0.016.

Quantitative dosing experiments were then performed using the previously characterised standard solution of approximately 3 nM ± 0.5 nM Aβ_42_ oligomers with approximately 500 nM Aβ_42_ monomer (Fig. 4). This standard solution was made freshly each day and then diluted to obtain different oligomer concentrations in the nanopipette. We have previously shown that the oligomers are stable for several hours at picomolar concentrations once formed. This allows us to have a defined concentration of oligomers in the pipette for dosing experiments, since there is no dissociation of the oligomers on dilution^20^. Using our model we can estimate the oligomer concentration at the cell surface. Under the conditions of the experiment the oligomer concentration on the cell surface, *c*_*surface*_, at the point under the centre of the pipette is 0.33*c*_*0*_, where *c*_*0*_ is the oligomer concentration in the pipette (see Supplementary materials). The oligomer concentration on the surface drops to 0.5*c*_*surface*_, at a radial distance of approximately 500 nm away from this centre point^19^. The average surface oligomer concentration in this area of 500 nm radius is 0.2*c*_*0*_, and this value will be quoted in the rest of the paper. When there are 30 pM oligomers in the pipette then we estimate that during the 10 minute experiment about 1250 oligomers were delivered to the cell surface, corresponding to 2 oligomers per second (see Supplementary materials for details). This means that it can be assumed that individual oligomers are interacting with the cell surface one by one during the experiment. The average oligomer surface concentration, in the area below the pipette, can similarly be estimated to be of the order of 5 pM. This concentration of oligomers is within an order of magnitude of the oligomer concentration to that estimated to be present in human CSF in patients with Alzheimer’s disease, 0.5 pM^12^, and gives a detectable but small increase in intracellular calcium.

The calcium influxes observed are complex since the cells can respond to the oligomer induced calcium influx over the 10 minute experiment and there was significant variation between the responses of different cells. We analysed these data by firstly looking at the average behaviour of all the cells dosed at a specific oligomer concentration at the cell surface. The averaged cellular response showed a steep increase in the beginning (first 100 seconds) of the experiments, increasing with the oligomer concentration at the cell surface, and then the calcium level plateaus or is reduced at later times as the cell responds to the rise in intracellular calcium (Fig. 3). We then integrated the normalised calcium influx for each cell over the 10 minute experiment and averaged these together to compare the total amount of calcium entering the cell at a specific oligomer concentration at the cell surface (Fig. 4A). The total calcium entering the cell increases with oligomer concentration until at high oligomer concentrations the cell responds by reducing the intracellular calcium. Due to the fact that the cells respond to reduce oligomer induced calcium influx, the overall increase in total calcium is not proportional to the oligomer concentration, so that a 100 fold increase in oligomer surface concentration from 5 to 500 pM only doubles the total amount of calcium entering the cell. At low surface oligomer concentrations, 5 pM, there is still a statistically significant increase in the total amount of calcium over 10 minutes compared to the control (p-value of 0.0197). To confirm that the effects we observed are due to oligomers we performed dosing experiments in the presence or absence of clusterin, an extracellular chaperone that we have shown to preferentially bind oligomers^13^,^23^ (Fig. 4B–D). No significant calcium influx was observed in the presence of clusterin confirming that Aβ_42_ oligomers are causing the influx.

**Figure 5:**
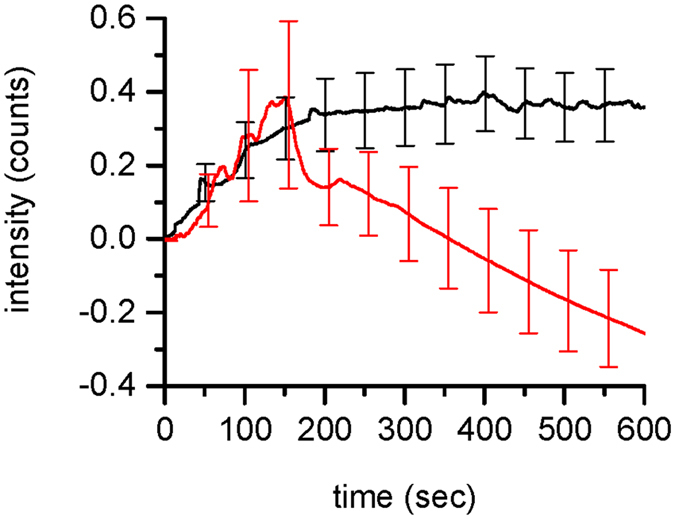
Recovery of astrocytes intracellular calcium after the application of Aβ_42_ oligomers. Cells were treated with an average surface concentration of 1250 pM Aβ_42_ oligomers for 10 minutes in the control experiments or for the first 5 minutes and then observed for a further 5 minutes to determine if the cells recovered. The traces are the average of the response from several individual cells (n = 10 control, n = 3 recovery). The error bars, SEM, are shown in both traces every 50 seconds.

To test whether cells were still able to recover after the application of Aβ_42_ oligomers the pipette was removed after 5 minutes from the cell when dosing with a surface concentration of 1250 pM oligomers. The results were compared to experiments with application over the full 10 minutes. Figure 5 shows that astrocytes were indeed able to remove the excess calcium and recover back to basal levels. This suggests that any membrane disruption or pore formed by the oligomers is transient. If permanent pores were formed in the cell membrane then the calcium influx would be expected to continue to increase when dosing of oligomers was stopped.

### Experiments on neurons

**Figure 6:**
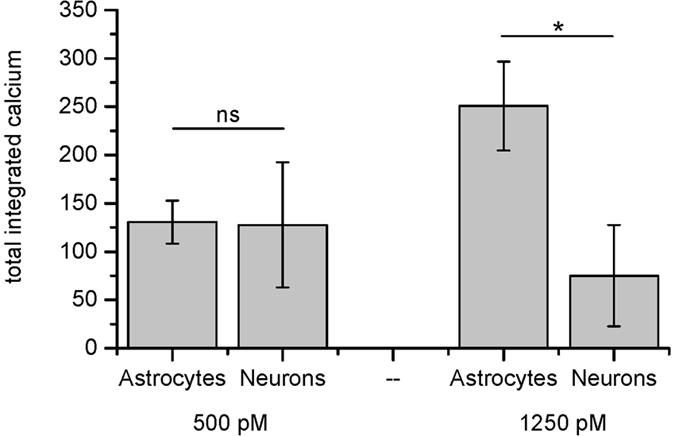
Comparison of the integrated change in intracellular calcium over 10 minutes between astrocytes (n = 6 and n = 14) and neurons (n = 3 and n = 7) at oligomer surface concentrations of either 500 or 1250 pM respectively, corrected for photobleaching and dye loss. Error bars are SEM. The p values were 0.9995 for the 500 pM data and 0.0281 for the 1250 pM data, using a two way ANOVA, showing that there is a significant difference in the oligomer dose response of astrocytes and neurons. The normalised intracellular calcium traces for neurons are shown in Supplementary Figure S3.

We next repeated these experiments on neurons at a surface oligomer concentration of 500 and 1250 pM (Fig. 6 and Supplementary Figure S3). Neurons showed a similar response to astrocytes at 500 pM but a significantly smaller response at 1250 pM, due to the neurons responding to the dosed oligomers by reducing the intracellular calcium. These data suggest that Aβ_42_ oligomers have similar effects on astrocytes and neurons but that the cells respond differently to the oligomer induced calcium influx and that neurons respond to reduce the intracellular calcium at a lower oligomer concentration.

### Inhibition of calcium influx with nanobodies

**Figure 7:**
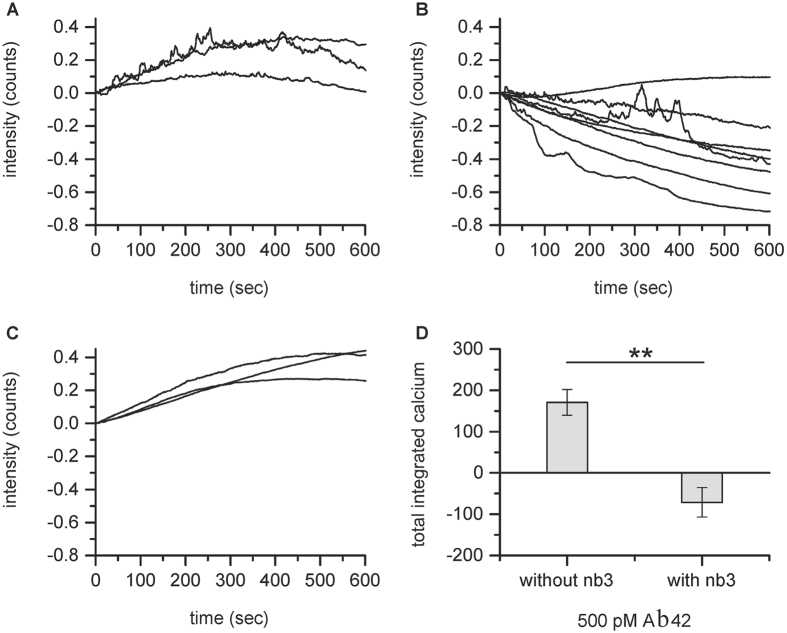
(**A**) Astrocytes were dosed with an average surface concentration of 500 pM Aβ_42_ oligomers (n = 3) and showed calcium entry. (**B**) Astrocytes were dosed with an average surface concentration of 500 pM Aβ_42_ oligomers after pre-incubation with 150 nM Nb3, a nanobody to Aβ (n = 8). The concentration of oligomers during the pre-incubation is approximately 3 nM and monomer concentration is approximately 500 nM. Blocking of calcium entry was observed. (**C**) Astrocytes were dosed with an average surface concentration 500 pM Aβ_42_ oligomers after pre-incubation with 150 nM of an alpha synuclein specific nanobody, NbSyn87 (n = 3). The oligomer concentration during the pre-incubation is approximately 3 nM and monomer concentration is approximately 500 nM. No blocking of calcium entry was observed. (**D**) Average integrated calcium in the presence and absence of the nanobody to Aβ, corrected for photobleaching and dye loss. Error bars are SEM. The p value was 0.0013.

One advantage of our approach is that it can be used to test the effectiveness of antibodies or nanobodies that target Aβ at reducing oligomer induced calcium influx. We used a nanobody, Nb3, that binds to the epitope 17–28 of Aβ with a measured K_d_ for the monomer of 13 nM^24^. In these experiments we pre-incubated synthetic oligomers of Aβ_42_ with 150 nM Nb3 for 15 minutes and then performed nanopipette dosing experiments on astrocytes (Fig. 7). Our experiments show that Nb3 is very effective at blocking the calcium influx caused by Aβ_42_ oligomers. This reduction in calcium influx was presumably due to nanobodies binding to the oligomers, reducing or preventing interaction with the cell membrane.

### Bilayer experiments

**Figure 8:**
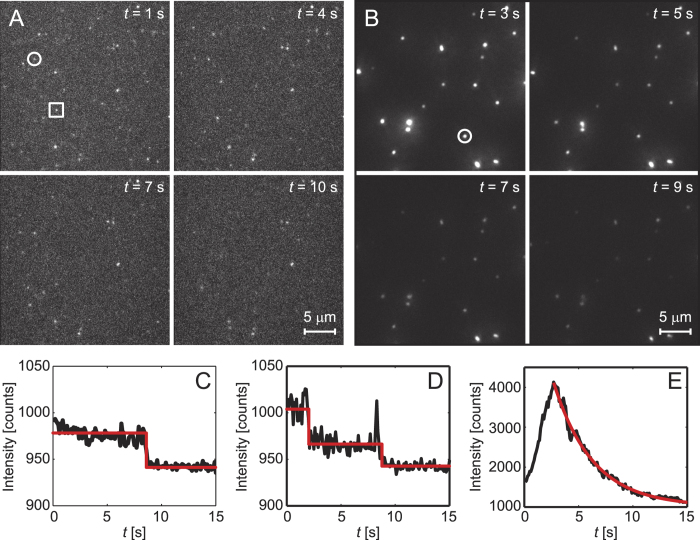
Fluorescence microscopy images showing Aβ_42_-Alexa 647 monomers and oligomers immobilised on (**A**) a glass surface and (**B**) a POPC (uncharged) lipid bilayer at four consecutive times. (**C–D**) Fluorescence intensity traces showing single and two-step photobleaching for two different Aβ_42_-Alexa 647 molecules on the glass surface corresponding to an Aβ monomer (circle in **A**) and dimer (square in **A**), respectively. (**E**) Fluorescence intensity trace showing an exponential decay in the fluorescence signal from an Aβ_42_-Alexa 647 larger oligomeμr in the lipid bilayer (circle in **B**).

To further investigate the molecular mechanism of cellular damage we performed experiments on model bilayers. Fluorescently labelled Aβ_42_ was added to either a glass slide or to a supported lipid bilayer (SLB), using solutions prepared from the same stock solution, to further elucidate the mechanism by which Aβ inserts or crosses the cell membrane (Fig. 8A,B). An Aβ_42_ monomer bath concentration of 2.5 nM with 15 pM oligomers was used for the glass slide experiments, whereas a 100 times higher concentration was used for the SLB experiments, to compensate for Aβ_42_ binding less readily to the SLB. Single molecules could easily be discerned in both cases and oligomers were distinguished from monomers on the basis of their intensity and the number of steps in their photobleaching profiles. A monomer bleaches with a single step while a dimer bleaches in two steps etc (Fig. 8C,D). Molecules with four or more bleaching steps approach an exponential bleaching profile and are here termed “tetramers or larger” oligomers (Fig. 8E).

The total amount of bound Aβ_42_ on the glass slide was 1.4 times higher than on the SLB. For the glass surface we counted 77 ± 3% monomers, 16 ± 3% dimers, 2 ± 1% trimers and 6 ± 2% tetramers or larger (mean value ± one standard deviation estimated from a binomial distribution of n = 174 molecules). These percentages are likely different from our confocal measurements in solution due to the tetramer or larger oligomers binding preferentially to the glass, as observed previously^20^. However, whereas only a smaller fraction of the molecules on the glass surface were tetramers or larger oligomers, we found that more than 90 ± 3% of the molecules on the lipid bilayer were tetramers or larger oligomers (mean value ± one standard deviation estimated from a binomial distribution of n = 128 molecules). That the SLB has a higher fraction of tetramers or larger oligomers than glass is statistically significant based on a two-proportion z-test (p-value of 10^−49^). In fact, the actual number of tetramer or larger oligomers on the SLB is estimated to be close to 100% when the binding of Aβ_42_ to defects in the lipid bilayer is taken into account. This was estimated by counting the number of monomers to trimers on the SLB at different concentrations of Aβ_42_ in the bath solution. Aβ_42_ was observed to bind more rapidly to glass than the SLB, so that the Aβ_42_ will first bind to holes/defects in the SLB. Using photobleaching step analysis the numbers of monomers to trimers were found to remain approximately the same when the concentration was changed from 2.5 nM to 250 nM (between 5% and 13% of the total numbers of molecules in the 250 nM sample), whereas the concentration of tetramer or larger oligomers increased monotonically with total protein concentration. Since there are two orders of magnitude less tetramer or larger oligomers than monomers in the bath solution, this also explains why a higher bath concentration of Aβ_42_ is needed for the lipid bilayer experiments to get comparable numbers of bound molecules, as for the glass slide experiments. It should also be noted that the initial increase in intensity seen in Fig. 8E is not due to addition of Aβ_42_ from solution since the bath solution was replaced with buffer solution without Aβ_42_, but is a phenomenon we see for all tetramer or larger oligomers in the SLB. This is possibly due to photoisomerisation of Alexa-647 from dark states to a bright trans state, when excited by the laser^25^.

From the SLB experiments we could furthermore distinguish between whether the oligomers tend to (i) bind on top of the lipid bilayer or whether they (ii) insert or try to cross the SLB. In the former case we expect any bound Aβ_42_ to be mobile since the lipids the Aβ_42_ bind to are mobile. However, the majority of the bound Aβ_42_ molecules on the SLB do not change their position noticeably and also remain in the same position after the bulk solution is exchanged with buffer solution (see Fig. 8 and Supplementary Movie 2). This suggests that either the Aβ_42_ inserts fully into the SLB, which happens on a timescale <1 s based on the frame rate of these experiments, or tries to cross the lipid bilayer. If the Aβ_42_ spans the entire SLB, or tries to cross it, then the close proximity to the glass support will make Aβ_42_ stick to the surface. The majority of the tetramer or larger oligomers appear to fully insert into or try to cross the uncharged lipid bilayer in contrast to Aβ_42_ monomers that did not significantly interact with the uncharged lipids.

Overall these data show that tetramers or larger oligomers can span or cross lipid bilayers, a result consistent with our previous observation that oligomers can enter cells at 4 °C^26^, indicating that no active process is required.

## Discussion

We used SICM dosing in these experiments since we reasoned that more oligomers would reach the cell surface per second by local dosing via the pipette than by bath application, so that calcium influx should be observed at lower surface concentrations. The reason for this is due to a depletion layer of oligomers close to the cell surface in bath application, which is reduced when delivering via the pipette close above the cell surface. This was confirmed by our experiments. In the brain the area of interaction of CSF with the cell is increased by a factor of 10–100, compared to dosing with a pipette, and the gap between cells is only 10–20 nm^27^. Higher number of oligomers would therefor encounter a cell each second and more calcium influx would occur. However, an additional factor in the brain is that in the extracellular fluid there is already a significant concentration of extracellular chaperones that can bind to the oligomers and prevent calcium influx. If, for example, 90–99%, of the oligomers are sequestered, then the concentration of unsequestered oligomers is only 1–10% of the total oligomer concentration. The net result should be that the oligomer induced calcium influx experienced by a cell in the brain is within a factor of 1–10 of the influx experienced by a cell in our vitro experiments, using local application via a pipette. Therefore we are working in an experimental regime where we observed the initial effects of oligomers on neuronal cells. An additional advantage of using SICM dosing is that only small volumes of solution are needed, 10 μL, allowing the straightforward extension of these experiments to dosing with samples of human CSF from AD patients in future studies.

While previous work has suggested that the initial damage caused by Aβ oligomers occurs at astrocytes^14^,^20^,^28^ not neurons, our work shows that calcium influx can occur at average concentrations of oligomers on the cell surface down to 5 pM, comparable to the concentration of oligomers reported in human CSF, and occurs in both neurons and astrocytes. However, there are still some significant differences between the two experiments. In this work we have blocked oscillations induced by mGlu5 in astrocytes, allowing us to detect the slow rise in calcium influx in both neurons and astrocytes. In addition, we have a much higher rate of encounter between the oligomers and the cell surface, using nanopipette dosing, which more closely represents the situation in the brain, so we can observe detectable changes at lower oligomer concentrations. Our work shows that the cell responds to the calcium influx so that there is an increase in the total amount of calcium entering the cell as the oligomer concentrations is increased, but that this is not linear with oligomer concentration. At a threshold intracellular calcium concentration the cell appears to respond to the calcium influx and reduce the intracellular calcium and this threshold is lower for neurons than astrocytes.

Our data are consistent with the calcium influx occurring as the individual oligomers cross the cell membrane, so that when dosing is stopped the calcium influx is stopped and the cells can reduce the calcium to basal levels. During dosing the rate of calcium entry is presumably faster than the rate at which the cell can remove calcium, leading to an overall increase but, when dosing stops, the cell can recover. It is also worth noting that the calcium influxes observed are small, corresponding to less than 10% of the full dynamic range of Fluo-4 when saturating amounts of calcium are used. Thus the oligomers induce a low level “calcium trickle” over 10 minutes rather than a rapid and large change in calcium, which would lead to rapid calcium removal by calcium pumps. This low level of calcium entry was not detectable in our previous experiments due to the calcium oscillations. In the brain, where no mGlu5 blocker is present, we expect this low level oligomer induced calcium increase to occur in addition to calcium oscillations in astrocytes. This would result in oscillations of other coupled astrocytes over wider areas creating additional problems in calcium homeostasis. The longer term effect of these astrocyte oscillations, as shown in other work, could be transcriptional changes and remodelling of the calcium signalling tool-kit of astrocytes that is selective to different/certain brain regions, providing an explanation of why certain brain regions are more prone to damage in Alzheimer’s disease^29^. There has also been one experiment performed on Aβ oligomer induced blocking of long-term potentiation, which was prevented by the addition of a blocker of mGlu5^30^, although much higher oligomer concentrations were used in these studies. This suggests that the oligomer induced calcium oscillations may play an important role in the development of Alzheimer’s disease and may significantly amplify the effect of individual oligomers on astrocytes.

The number of any specific receptors in the 1 μm^2^ region of the cell membrane, dosed by the pipette, is small. The affinity of the oligomer for this receptors also needs to be in the picomolar range for any binding to occur when dosed with picomolar concentrations of oligomers. Furthermore, if the affinity of the oligomer for the receptors was in the picomolar range then the off-rate would be expected to be very slow. This is inconsistent with our finding that the cells can recover within 10 minutes. Therefore we conclude that the calcium influx is caused by individual oligomers crossing the cell membrane, without any interaction with a specific receptor. However based on our work we cannot totally discount the possibility of a specific interaction with a receptor occurring. Previous work also supports the conclusion that no receptor is involved. Oligomers of Aβ_42_ are ThT active^21^, presumably due to their beta sheet structure, and hence have hydrophobic groups pointing into solution, which is why tetramers or larger oligomers cross the lipid bilayer in our experiment, and also why they will bind and cross the cell membrane. A previous study has found small oligomers bound to the cell membrane at near physiological concentrations of Aβ monomer^31^. Studies of the permeabilization of liposomes, at much higher concentrations of Aβ oligomers, also show that no interaction with any receptor is required for molecules to exit the liposome^32^,^33^.

Our experiments do not allow us to determine the physical mechanism by which calcium enters the cell from the external media. This could be due to local disruption of the cell membrane or calcium ion entering at the same time that the oligomer crosses through the cell membrane. As calcium ions enter the cell, there will also be leakage of ATP from the cell into solution by the same mechanism. ATP leakage has been observed in other experiments at much higher acute oligomer concentrations^34^, and may affect neighbouring cells leading to the release of pro-inflammatory cytokines. The amount of calcium influx occurring as the oligomer crosses will potentially depend on the oligomer size. While our study cannot address this point, there has been a previous study on entry of Aβ oligomers into oocytes using fluorescence imaging to detect the calcium influx^35^. Oocytes have no calcium pumps, making the calcium entry easier to detect. These data were analysed as if the oligomers formed permanent pores in the cell membrane that opened and closed. In the light of our experiments these data can be reinterpreted as different oligomers entering the cell by crossing at certain regions of the cell membrane and the wide range of different open times observed can be explained by the different transit times of the oligomers through the cell membrane. These transit times were fitted by a double exponential function with time constants of 5 and 16 ms and estimated conductance of 0.4–4 picosiemens. A small fraction of the oligomers, presumably large oligomers, were found to take longer to cross the cell membrane and allow more calcium to enter the cell. This reinterpretation of the data is consistent with our finding that only about 1 in 500 Aβ oligomers were capable of triggering astrocyte oscillations^20^, presumably due to inducing a larger calcium influx. This could be due to the oligomer size or the composition of the membrane to which the oligomer binds or a combination of these two effects.

It is widely recognised that altered calcium homeostasis is a key feature of Alzheimer’ disease. The fact that the calcium influx depends on the number of individual oligomers encountering the cell membrane means that the amount of disruption to calcium homeostasis that a cell will experience depends on the number of unsequestered oligomers in the CSF. Higher concentrations of oligomers or lower concentrations of extra-cellular chaperones will result in a larger disruption to calcium homeostasis in some neuronal cells, particularly astrocytes. This will ultimately lead to altered cell physiology and cell death and also adversely affect neighbouring neurons. This may explain why small increases in oligomer concentrations can be significant over long timespans. Furthermore, the effect of oligomer induced calcium influx in a fine structure, such as a synapse or fine protrusions, will be much more significant than at the cell body due to the smaller volume. This provides a possible explanation for the detrimental changes oligomers cause at the synapses of neurons at low concentrations, without the need for any specific interactions with any receptors.

## Conclusions

We have developed a method to study the initial events that lead to cellular damage in Alzheimer’s disease. Our experiments suggest that as tetramers or larger oligomers cross the cell membrane of astrocytes or neurons, calcium enters. This calcium influx can be prevented by clusterin and by a nanobody targeting the mid-region of Aβ. There appears to be no receptor involved in these initial events. The presence of low picomolar concentrations of Aβ aggregates therefore leads to continual low levels of calcium influx altering calcium homeostasis. An increase in the number of aggregates or reduction in the level of clusterin, which sequesters the aggregates, will lead to an increased rate of calcium influx and consequential cellular damage and, over time, earlier disease onset.

## Material and Methods

### Cell culture

Astrocytes were from a rat mixed glial preparation, cultured for 14 days in 75 cm^2^ flasks with Dulbecco’s Modified Eagle’s (DMEM) Medium (Invitrogen) supplemented with 10% fetal bovine serum, 1% Penicillin and Streptomycin and 1% L-Glutamine (Life Technologies) in a humidified atmosphere containing 5% CO_2_ at 37 °C. Cells were passaged 2–3 times a week. For experiments the astrocytes were cultured to 40–70% confluence in 35 mm culture dishes with 14 mm glass microwell, number 1 thickness cover glass (MatTek). Experiments were performed 1–5 days after plating. Cells were loaded with 2.3 μM Fluo-4 AM (Life Technologies) for 10–15 minutes and washed twice with L15 (Life Technologies) afterwards.

### Aβ_42_ aggregation

HiLyte Fluor 647 Aβ_42_ (Cambridge Bioscience LDT) was purified on a Biosep SEC-s2000 size exclusion column (Phenomenex) using pH 7.4 SSPE (0.01 M Na_2_HPO_4_, 0.15 M NaCl, 1 mM EDTA) as the running buffer. The peptide was kept in ice prior to purification, flash frozen immediately after and stored at −80 °C. For the cell experiments, the purified Aβ_42_ was diluted to 500 nM in PBS and left shaking at 37 °C, 200 rpm for 5 hours. It was centrifuged at 14,500 g for 10 minutes and then diluted to the required concentration in L15 medium. Clusterin was a gift of Professor Mark Wilson at the University of Wollongong and prepared using published proceedures^36^. The clusterin was used at with 2 μg/mL.

### Aβ nanobody

The Aβ specific nanobody Nb3 was isolated from a llama (*Llama glama*) immunized with Aβ_40_ and by the construction and selection of a phage-display library containing the VHH genes, amplified from the peripheral blood lymphocytes, as described previously^37^. The Nb3 gene was subsequently recloned in a modified pHEN vector (pHEN6) containing a sequence coding for six consecutive histidine residues at the C-terminus of the nanobody and the nanobody was subsequently expressed in the periplasm of *Escherichia coli* and purified using immobilized metal affinity chromatography and size-exclusion chromatography, according to published protocols^38^. The concentration of Nb3 was measured by UV absorbance spectroscopy using a molecular extinction coefficient, which was calculated based on the sequence of the protein at 280 nM of 21,555 M^−1^ cm^−1^. The K_d_ of Nb3 for monomeric Aβ40 has previously been measured as 13 nM using a Biacore 3000 24.

### Synuclein nanobody

The α-synuclein specific nanobody, NbSyn87, was previously isolated by phage display selection following the immunization of a llama (*Llama glama*) with the A53T variant of the protein human α-synuclein^39^. The nanobody was subsequently recloned in a pHEN6 vector, expressed and purified according to an identical protocol as described above for the expression and purification of Nb3. The protein concentration of NbSyn87 was estimated by UV absorbance spectroscopy using a molecular extinction coefficient, calculated from the sequence of the protein, at 280 nm of 26,025 M^−1^ cm^−1^.

### Single molecule confocal set-up

The same set-up and method was used to determine the oligomer concentration as previously described^20^,^23^.

### ThT imaging

The protein was aggregated as described above. ThT (Sigma-Aldrich) stock solution was prepared in DMSO (Sigma-Aldrich) at a concentration of approximately 1 M and diluted into pre-filtered PBS (0.02 μm filter, Whatman) to a final concentration of ~100 μM. The final concentration was determined from the absorbance at 412 nm with an extinction coefficient of 36,000 M^−1^ cm^−1^. The stock solution was prepared freshly and filtered (0.02 μm filter, Whatman) on a daily basis. Borosilicate glass coverslips (VWR International) were cleaned in an argon ion plasma for 1 hour and prepared for coating with a 20 mm × 20 mm Frame-Seal slide chamber (Bio-rad). The coverslips were coated with Poly-(L)-Lysine (PLK) (Sigma-Aldrich) for at least one hour. The PLK-coated surfaces were washed three times with PBS before the sample was applied. Aggregated Aβ_42_ was used at a total monomer concentration of 1.25 nM in PBS with 5 μM ThT. Each sample was left on the coverslip for 10 minutes prior to imaging to ensure binding of the aggregates to the surface. The imaging was then performed with 5 μM ThT in PBS. The samples were imaged using a home-built total internal reflection fluorescence (TIRF) microscope limiting the detectable fluorescence signal to within 200 nm from the coverslip. A 405 nm Laser (LBX-LD, Oxxius Laser Box) was aligned and directed parallel to the optical axis at the edge of a TIRF objective (APON60XO TIRF, Olympus) mounted on an inverted Olympus IX-73 microscope for ThT imaging. Fluorescence was collected by the same objective, separated from the returning TIRF beam by a dichroic (Di01-405/488/532/635, Semrock), and passed through an appropriate filter (BLP01-488R-25, Laser 2000). The images were recorded on an EMCCD camera (Evolve 512 Delta, Photometrics) operating in frame transfer mode (EMGain of 4.4 e^−^/ADU and 250 ADU/photon). Each pixel was 207 nm. The distance between the 9 images measured in each grid was set to 350 μm, and was automated (bean-shell script, Micromanager) to prevent user bias. Images were recorded continuously for 100 frames with 50 ms exposure in the blue channel (ThT emission) with 405 nm illumination.. Images were recorded at 50 ms exposure with 100 frames for each field of view in the blue channel (ThT emission). Data analysis was done using custom written procedures using the GDCS SMLM plugin (Sussex University) for ImageJ (http://imagej.nih./goc/ij). Briefly, the image stacks were averaged over the full set and the background was removed and the 2D Gaussian point-spread-functions (PSF) were fitted using PeakFit. The photon values for the histogram were calculated using EMGain from the extracted intensities.

### SICM set-up

The SICM was built on an inverted Nikon Eclipse Ti-U. The microscope objective (Nikon, Plan Apo VC Water immersion ×60, MRD07601) was mounted in a piezoelectric drive (P-726, Physik Instrumente, UK) for this purpose, while the sample and nanopipette were positioned by XY (P-733.2DD) and Z (P-753) piezos, respectively. All piezos were controlled via a computer using an Ionscope controller.

Nanopipettes were fabricated using a Sutter Instrument Co. model P-2000 laser-based puller, pulling 10 cm length, 1 mm/0.50 mm outer/inner diameter fire-polished borosilicate glass capillaries with filaments (Sutter, via Intracel UK Ltd.) using the program Heat 350, Fil 3, Vel 30, Del 220, Pul, Heat 390, Fil 3, Vel 40, Del 180, Pul 255. The pull time range is 4.5–5.5 s. Typical nanopipette ion currents were 500 ± 1.5 pA pA for a 200 mV electrode bias. Calcium influx was monitored using an epifluorescence set-up using 488 nM laser excitation and an EMCCD camera (iXon DU 897, Andor) for detection.

### Calcium imaging experiments

For these experiments cells were kept in L15 (Life Technologies) containing 1.26 mM Ca^2+^. 300 μM 2-Methyl-6-(phenylethynyl)pyridine (MPEP) (Sigma Aldrich), with a specific metabotropic glutamate receptor 5 (mGlu5) blocker, was added to the bath to prevent calcium oscillations. The pipette contained L15 (Life Technologies) plus different concentrations of Aβ_42_ oligomers, with a total volume of 10 μL. 2 μM Ionomycin (Sigma Aldrich) added to the L15 for the positive control experiments. The computer-controlled nanopipette, mounted to the z-piezo, was positioned 300 nm above the cell. This distance was chosen by experience. It was far enough away to avoid mechanical stress to the cell, by activating mechanosensitive ion channels, and close enough to apply sufficient concentration to the cell. The pipette was kept in position using distance feedback control. Aβ_42_ was released from the pipette by applying −200 mV and 15 kPa pressure to the pipette. We chose a negative voltage because of the fact that HiLyte 647 has a slight negative charge. After the pipette was in place over the cell, calcium imaging was started and pressure applied to start delivery. The maximum observed fluorescence signals were about one order of magnitude smaller than the full dynamic range of Fluo-4 and are thus expected to be linearly proportional to Ca^2+^ flux. The data was normalised to the initial calcium fluorescence level, ΔF/F_o_, to take account of variation in Fluo-4 loading between cells. The normalised calcium traces were analysed using OriginPro 2015.

### Bilayer experiments

The SLB consisted of 1 palmitoyl-2-oleoyl-sn-glycero-3-phosphocholine (POPC) lipids from Avanti Polar Lipids with 0.01 wt% of the fluorescently labelled lipid Oregon Green^®^ 488 1,2-Dihexadecanoyl-sn-Glycero-3-Phosphoethanolamine (Oregon Green^®^ 488 DHPE; Invitrogen, UK) to visualize the SLB in order to make sure there were no defects. After forming the SLB, Aβ labelled with HiLyte 647 was added to the dish. This was done at different concentrations (higher concentration leads to more binding), all in L15 media (from 2.5 nM to 250 nM). The binding of the Aβ was imaged with TIRF microscopy (with a HeNe laser operating at 633 nM) either with Aβ in the solution (at 1 s between frames) or after rinsing the solution to remove unbound Aβ (stream acquisition with 100 ms exposure time to measure single-step photobleaching).

### Statistical analysis

Analyses of differences between two groups were conducted using a standard unpaired Student’s *t* test. For the data in Fig. 6 a two way ANOVA test (Sidak’s multiple comparison test) was performed.

## Additional Information

**How to cite this article**: Drews, A. *et al.* Individual aggregates of amyloid beta induce temporary calcium influx through the cell membrane of neuronal cells. *Sci. Rep.* **6**, 31910; doi: 10.1038/srep31910 (2016).

## Supplementary Material

Supplementary Movie 1

Supplementary Movie 2

Supplementary Information

## References

[b1] Giannakopoulos P. *et al.* Quantitative analysis of tau protein-immunoreactive accumulations and beta amyloid protein deposits in the cerebral cortex of the mouse lemur, Microcebus murinus. Acta Neuropathol. 94(2), 131–139 (1997). 9255387 10.1007/s004010050684

[b2] Masters C. L. *et al.* Neuronal origin of a cerebral amyloid: neurofibrillary tangles of Alzheimer’s disease contain the same protein as the amyloid of plaque cores and blood vessels. EMBO 4(11), 2757–2763 (1985). 10.1002/j.1460-2075.1985.tb04000.x PMC554575 4065091

[b3] Näslund J. H. V. *et al.* Correlation between elevated levels of amyloid beta-peptide in the brain and cognitive decline. JAMA. 283(12), 1571–1577 (2000). 10735393 10.1001/jama.283.12.1571

[b4] Shankar G. M. *et al.* Amyloid-beta protein dimers isolated directly from Alzheimer’s brains impair synaptic plasticity and memory. Nat Med 14, 837–842, doi: 10.1038/nm1782 (2008). 18568035 PMC2772133

[b5] Bucciantini M. *et al.* Inherent toxicity of aggregates implies a common mechanism for protein misfolding diseases. Nature 416, 507–511 (2002). 11932737 10.1038/416507a

[b6] Shankar G. M. *et al.* Amyloid-[beta] protein dimers isolated directly from Alzheimer’s brains impair synaptic plasticity and memory. Nat Med 14, 837–842, doi: http://www.nature.com/nm/journal/v14/n8/suppinfo/nm1782_S1.html (2008). 18568035 10.1038/nm1782 PMC2772133

[b7] Chiti F. & Dobson C. M. Protein Misfolding, Functional Amyloid, and Human Disease. Annual Review of Biochemistry 75, 333–366, doi: 10.1146/annurev.biochem.75.101304.123901 (2006). 16756495

[b8] Kayed R. & Lasagna-Reeves C. A. Molecular mechanisms of amyloid oligomers toxicity. Journal of Alzheimer’s disease: JAD 33 Suppl 1, S67–78, doi: 10.3233/jad-2012-129001 (2013). 22531422

[b9] Masters C. L. & Selkoe D. J. Biochemistry of amyloid beta-protein and amyloid deposits in Alzheimer disease. Cold Spring Harbor perspectives in medicine 2, a006262, doi: 10.1101/cshperspect.a006262 (2012). 22675658 PMC3367542

[b10] Walsh D. M. *et al.* Naturally secreted oligomers of amyloid β protein potently inhibit hippocampal long-term potentiation *in vivo*. Nature 416, 535–539 (2002). 11932745 10.1038/416535a

[b11] Yerbury J. J. & Wilson M. R. Extracellular chaperones modulate the effects of Alzheimer’s patient cerebrospinal fluid on Aβ(1–42) toxicity and uptake. Cell Stress & Chaperones 15, 115–121, doi: 10.1007/s12192-009-0122-0 (2010). 19472074 PMC2866977

[b12] Savage M. J. *et al.* A sensitive aβ oligomer assay discriminates Alzheimer’s and aged control cerebrospinal fluid. The Journal of Neuroscience: The Official Journal of the Society for Neuroscience 34, 2884–2897, doi: 10.1523/JNEUROSCI.1675-13.2014 (2014). 24553930 PMC6608513

[b13] Narayan P. *et al.* Rare individual amyloid-β oligomers act on astrocytes to initiate neuronal damage. Biochemistry 53, 2442–2453, doi: 10.1021/bi401606f (2014). 24717093 PMC4004235

[b14] Abramov A. Y., Canevari L. & Duchen M. R. Calcium signals induced by amyloid β peptide and their consequences in neurons and astrocytes in culture. Biochimica et Biophysica Acta (BBA) - Molecular Cell Research 1742, 81–87, doi: 10.1016/j.bbamcr.2004.09.006 (2004). 15590058

[b15] Abramov A. Y., Canevari L. & Duchen M. R. Changes in intracellular calcium and glutathione in astrocytes as the primary mechanism of amyloid neurotoxicity. J Neurosci. 23(12), 5088–5095 (2003). 12832532 10.1523/JNEUROSCI.23-12-05088.2003 PMC6741151

[b16] Shevchuk A. I. *et al.* Simultaneous measurement of Ca2+ and cellular dynamics: combined scanning ion conductance and optical microscopy to study contracting cardiac myocytes. Biophysical Journal 81, 1759–1764 (2001). 11509385 10.1016/S0006-3495(01)75826-2 PMC1301650

[b17] Miragoli M. *et al.* Scanning ion conductance microscopy: a convergent high-resolution technology for multi-parametric analysis of living cardiovascular cells. J R Soc Interface. 8(60), 913–925 (2011). 21325316 10.1098/rsif.2010.0597 PMC3104336

[b18] Takahashi Y. *et al.* Multifunctional nanoprobes for nanoscale chemical imaging and localized chemical delivery at surfaces and interfaces.. Angew Chem Int Ed Engl. 50(41), 9638–9642 (2011). 21882305 10.1002/anie.201102796

[b19] Babakinejad B. *et al.* Local delivery of molecules from a nanopipette for quantitative receptor mapping on live cells.. Anal Chem. 85(19), 9333–9342 (2013). 24004146 10.1021/ac4021769

[b20] Narayan P. *et al.* Rare individual amyloid-β oligomers act on astrocytes to initiate neuronal damage. Biochemistry 53(15), 2442–2453 (2014). 24717093 10.1021/bi401606f PMC4004235

[b21] Horrocks M. H. *et al.* Single-molecule imaging of individual amyloid protein aggregates in human biofluids. ACS Chemical Neuroscience, doi: 10.1021/acschemneuro.5b00324 (2016). PMC4800427 26800462

[b22] Shevchuk A. I. *et al.* Imaging Proteins in Membranes of Living Cells by High-Resolution Scanning Ion Conductance Microscopy. Angewandte Chemie International Edition 45, 2212–2216, doi: 10.1002/anie.200503915 (2006). 16506257

[b23] Narayan P. *et al.* The extracellular chaperone clusterin sequesters oligomeric forms of the amyloid-β 1–40 peptide. Nature Structural and Molecular Biology 19, 79–84, doi: 10.1038/nsmb.2191 (2012). PMC4979993 22179788

[b24] Vincke C. Humanisation of Nanobodies and identification of Nanobodies for Alzheimer’s Disease immunotherapy. PhD thesis, Vrije Universiteit Brussel, Brussels, Belgium (2009).

[b25] White S. S. *et al.* Characterization of a single molecule DNA switch in free solution. Journal of the American Chemical Society 128, 11423–11432, doi: 10.1021/ja0614870 (2006). 16939265

[b26] Narayan P. *et al.* Single molecule characterization of the interactions between amyloid-β peptides and the membranes of hippocampal cells. Journal of the American Chemical Society 135, 1491–1498, doi: 10.1021/ja3103567 (2013). 23339742 PMC3561772

[b27] Syková E. & Nicholson C. Diffusion in Brain Extracellular Space. Physiological reviews 88, 1277–1340, doi: 10.1152/physrev.00027.2007 (2008). 18923183 PMC2785730

[b28] Garwood C. J., Pooler A. M., Atherton J., Hanger D. P. & Noble W. Astrocytes are important mediators of A[beta]-induced neurotoxicity and tau phosphorylation in primary culture. Cell Death and Dis 2, e167, doi: http://www.nature.com/cddis/journal/v2/n6/suppinfo/cddis201150s1.html (2011). 10.1038/cddis.2011.50 PMC3168992 21633390

[b29] Grolla A. A. *et al.* Amyloid-[beta] and Alzheimer/’s disease type pathology differentially affects the calcium signalling toolkit in astrocytes from different brain regions. Cell Death Dis 4, e623, doi: 10.1038/cddis.2013.145 (2013). 23661001 PMC3674354

[b30] Rammes G., Hasenjäger A., Sroka-Saidi K., Deussing J. M. & Parsons C. G. Therapeutic significance of NR2B-containing NMDA receptors and mGluR5 metabotropic glutamate receptors in mediating the synaptotoxic effects of β-amyloid oligomers on long-term potentiation (LTP) in murine hippocampal slices. Neuropharmacology 60, 982–990, doi: http://dx.doi.org/10.1016/j.neuropharm.2011.01.051 (2011). 21310164 10.1016/j.neuropharm.2011.01.051

[b31] Nag S., Chen J., Irudayaraj J. & Maiti S. Measurement of the Attachment and Assembly of Small Amyloid-β Oligomers on Live Cell Membranes at Physiological Concentrations Using Single-Molecule Tools. Biophysical Journal 99, 1969–1975, doi: http://dx.doi.org/10.1016/j.bpj.2010.07.020 (2010). 20858443 10.1016/j.bpj.2010.07.020 PMC2941002

[b32] Wong P. T. *et al.* Amyloid-β Membrane Binding and Permeabilization are Distinct Processes Influenced Separately by Membrane Charge and Fluidity. Journal of Molecular Biology 386, 81–96, doi: http://dx.doi.org/10.1016/j.jmb.2008.11.060 (2009). 19111557 10.1016/j.jmb.2008.11.060

[b33] Williams T. L., Day I. J. & Serpell L. C. The Effect of Alzheimer’s Aβ Aggregation State on the Permeation of Biomimetic Lipid Vesicles. Langmuir 26, 17260–17268, doi: 10.1021/la101581g (2010). 20923185

[b34] Sáez-Orellana F. *et al.* ATP leakage induces P2XR activation and contributes to acute synaptic excitotoxicity induced by soluble oligomers of β-amyloid peptide in hippocampal neurons. Neuropharmacology 100, 116–123, doi: http://dx.doi.org/10.1016/j.neuropharm.2015.04.005 (2016). 25896766 10.1016/j.neuropharm.2015.04.005

[b35] Demuro A., Smith M. & Parker I. Single-channel Ca2+ imaging implicates Aβ1–42 amyloid pores in Alzheimer’s disease pathology. The Journal of Cell Biology 195, 515–524, doi: 10.1083/jcb.201104133 (2011). 22024165 PMC3206345

[b36] Wilson M. R. & Easterbrook-Smith S. B. Clusterin binds by a multivalent mechanism to the Fcand Fab regions of IgG. Biochim. Biophys. Acta 1159, 319–326 (1992). 1390937 10.1016/0167-4838(92)90062-i

[b37] Paraschiv G. *et al.* Epitope structure and binding affinity of single chain llama anti-β-amyloid antibodies revealed by proteolytic excision affinity-mass spectrometry. Journal of Molecular Recognition 26, 1–9, doi: 10.1002/jmr.2210 (2013). 23280612

[b38] Conrath K. E. *et al.* β-Lactamase Inhibitors Derived from Single-Domain Antibody Fragments Elicited in the Camelidae. Antimicrobial Agents and Chemotherapy 45, 2807–2812, doi: 10.1128/AAC.45.10.2807-2812.2001 (2001). 11557473 PMC90735

[b39] Guilliams T. *et al.* Nanobodies Raised against Monomeric α-Synuclein Distinguish between Fibrils at Different Maturation Stages. Journal of Molecular Biology 425, 2397–2411, doi: http://dx.doi.org/10.1016/j.jmb.2013.01.040 (2013). 23557833 10.1016/j.jmb.2013.01.040

