# Supplementary material for: Individual aggregates of amyloid beta induce temporary calcium influx through the cell membrane of neuronal cells
Source: Sci Rep. 2016 Aug 24;6:31910. doi: 10.1038/srep31910 (PMC4995397; doi:10.1038/srep31910)
Supplement: Supplementary Information [file srep31910-s3.doc]

**Supplementary materials**

**Individual aggregates of amyloid beta induce temporary calcium influx through the cell membrane of neuronal cells**

Anna Drews, Jennie Flint, Nadia Shivji, Peter Jönsson, David Wirthensohn, Erwin De Genst, Cécile Vincke, Serge Muyldermans, Chris Dobson, David Klenerman

**Control experiments**


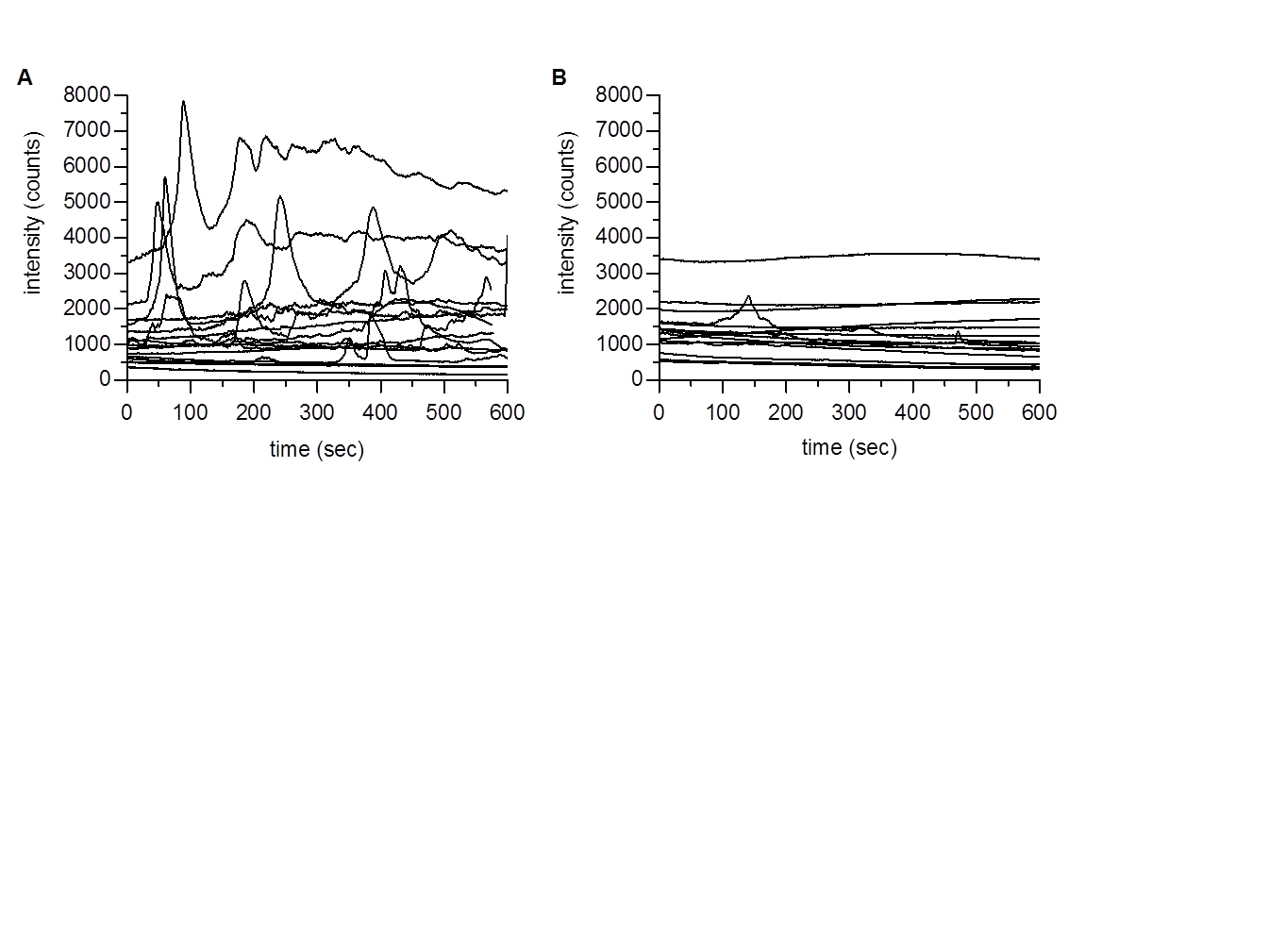


**Figure S1.** Calcium oscillations are blocked by MPEP. Multiple intracellular calcium traces for astrocytes without (A) (n = 17) and with 300 µM 2-Methyl-6-(phenylethynyl)pyridine (B) (n = 15) in the bath solution. Only the medium L15 was applied from the nanopipette in both (A) and (B).


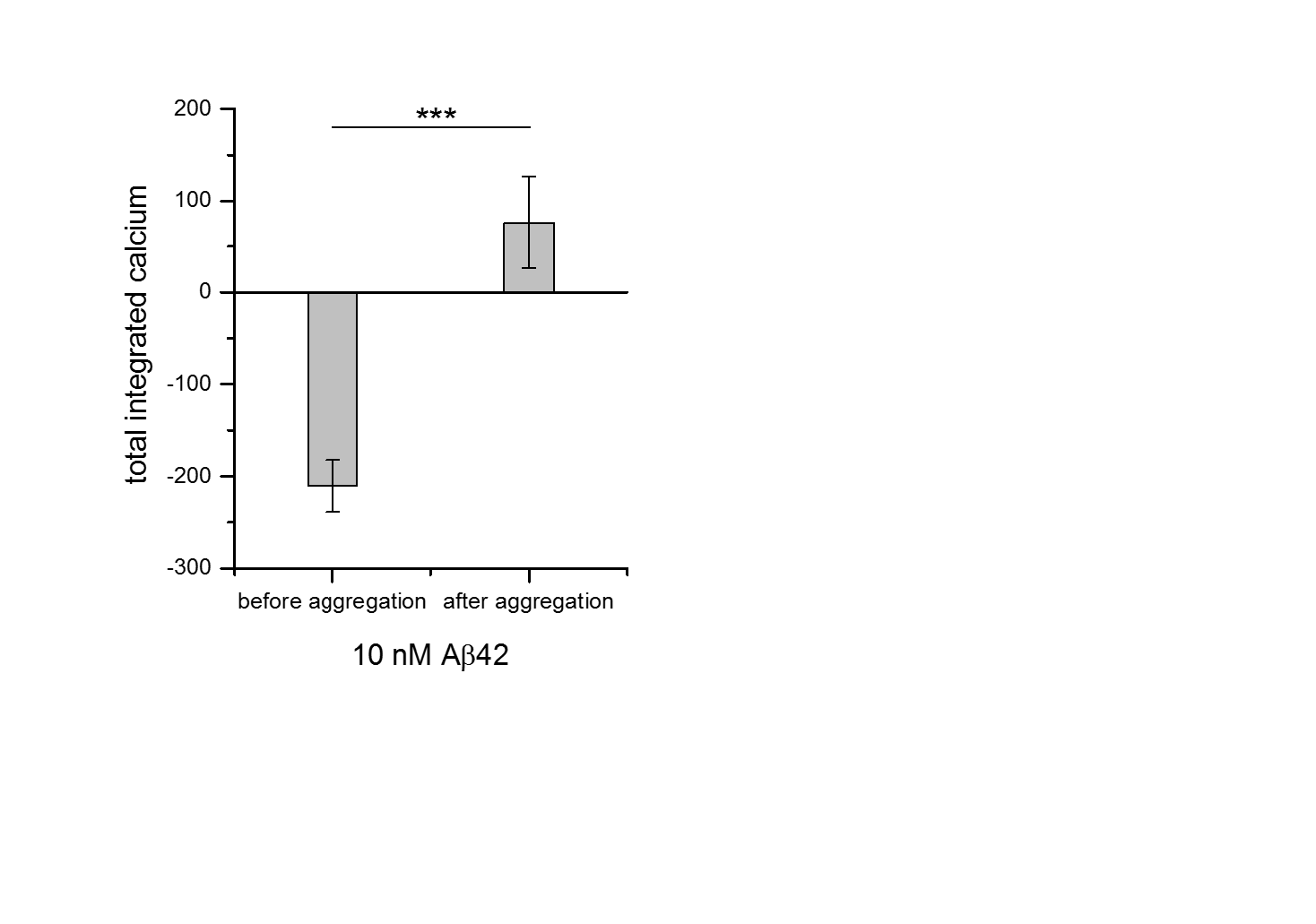


**Figure S2**. Nanopipette dosing before and after Aβ_42_ aggregation. 10nM Aβ_42_ monomer only, before aggregation (n=3), was dosed from the nanopipette onto individual astrocytes for 10 minutes and the total integrated calcium measured and corrected for photobleaching. The same experiment was performed on Aβ_42_ after aggregation, which produces 50 pM oligomer with 10 nM monomer (n=12). The error bars represent SEM. A two way t-test was performed with a p-value of 0.00029.


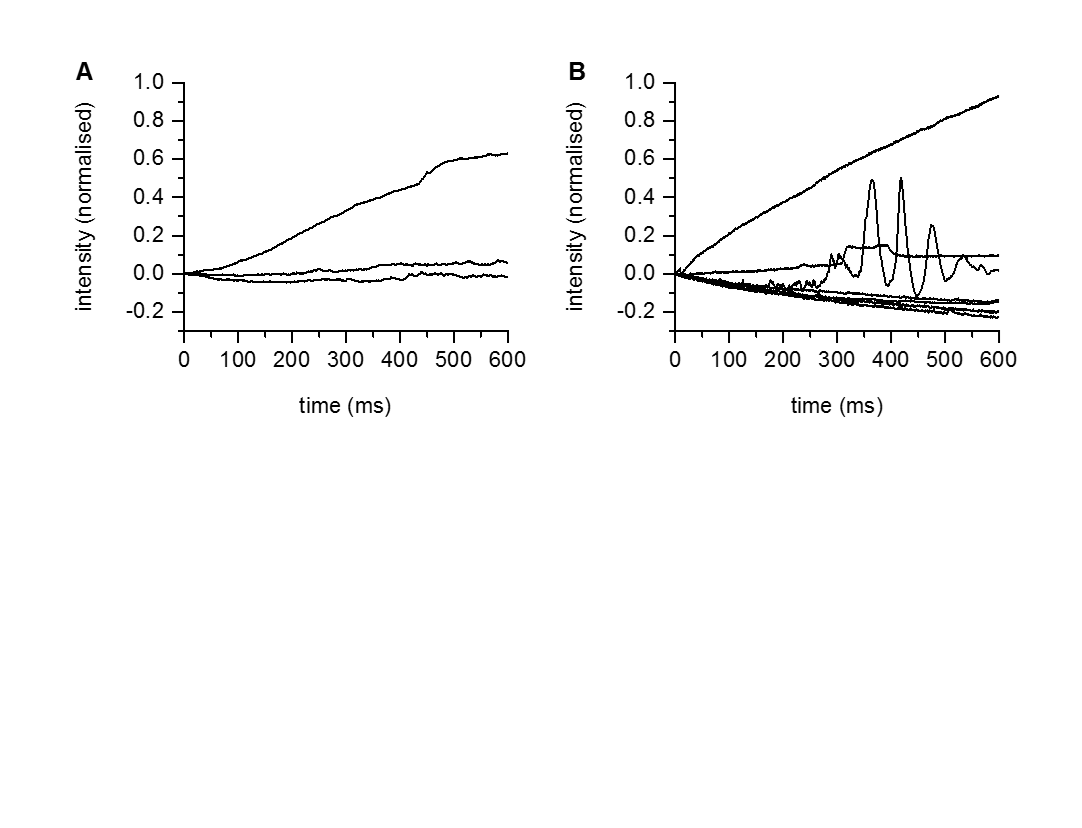


**Figure S3**. The change in the normalised intracellular calcium of neurons with an Aβ_42_ oligomer surface concentrations of A) 500 pM (n =3), B) 1250 pM. (n = 7).

**Table S1**

The n values for each trace in Figure 3.

| **Oligomer concentration** | **Neg. control** | **Pos. Control** | **500 fM** | **5 pM** | **50pM** | **500pM** | **1250 pM** | **1750 pM** | **2500 pM** |
| --- | --- | --- | --- | --- | --- | --- | --- | --- | --- |
| **n value** | 15 | 9 | 8 | 10 | 12 | 6 | 14 | 11 | 6 |

**Estimation of number of oligomers delivered**

The number of oligomers being delivered from the pipette (50 nm inner radius, inner cone angle of 3 degrees) is approximately:

*N*_tot_ = 1.16×10^-16^*c*_0_

for an applied pressure of 15 kPa over the pipette, where *c*_0_ is the number of oligomers in the pipette solution. For a solution with 1 nM oligomers in the pipette this means that *N*_tot_ = 70 oligomers/s. Since we are close to the cell (*h* = 300 nm away) most of these molecules will “hit” the cell surface. If only one hit is required for the oligomers to go into the cell (or bind to it) then the number of oligomers entering/binding to the cell per unit time will also be *N*_tot_. The majority of these oligomers will enter/bind to the cell just below the pipette (within a circular area with a radius of the order of the distance *h* between the pipette and the cell surface).

The amount of molecules leaving the pipette per time depends on the applied pressure, Δ*p*, and voltage, Δ*ψ*, over the pipette according to reference 19:

$N_{\text{tot}}=c_{0}Q_{\text{tot}}=c_{0}\left(\mu_{\text{ep}}+\mu_{\text{eo}}\right)\pi R_{0}\tan\left(\theta\right)\Delta\Psi+c_{0}\frac{3\pi R_{0}^{3}\tan\left(\theta\right)\Delta p}{8\eta}$ (1)

where *c*_0_ is the concentration of Aβ_42_ loaded in the pipette, *µ*_ep_ and *µ*_eo_ are the electrophoretic and electroosmotic mobility of Aβ_42_, respectively, *R*_0_ is the tip radius and *θ* is the inner half cone angle of the pipette and *η* the viscosity of the medium (1 mPa s). For a glass pipette in a 150 mM Na^+^ electrolyte *µ*_eo_ ~ 1.4×10^-8^ m^2^/V s [^1^](#_ENREF_1). The electrophoretic mobility of Aβ_42_ is harder to predict since it can be expected to depend both on the salt concentration in the medium and on the fluorescent labelling of the protein. For unlabelled Aβ_42_ in 10 mM TRIS the value of *µ*_ep_ has been measured to approximately -1×10^-8^ m^2^/V s for monomers and -2×10^-8^ m^2^/V s for aggregates [^2^](#_ENREF_2). For a pipette with *R*_0_ = 50 nm and *θ* = 3°, and with Δ*p* = 15 kPa and Δ*ψ* = -200 mV this gives *N*_tot_ = 1.09×10^‑16^*c*_0_ for monomers and *N*_tot_ = 1.26×10^-16^*c*_0_ for aggregates, but it should be noted that these values might be different under our experimental conditions. However, as an order of magnitude estimate we can approximate the delivery rate by setting Δ*ψ* = 0 which gives *N*_tot_ = 1.16×10^-16^*c*_0_ for an applied pressure of 15 kPa.

The concentration on the surface below the pipette can be determined from[^1^](#_ENREF_1) :

$c_{\text{surface}}=2c_{0}\left(1-\exp\left(-Q_{\text{tot}}/4\pi Dh\right)\right)$ (2)

where *D* is the diffusion coefficient of Aβ_42_ and *h* is the distance between the pipette and the cell surface. The diffusivity for Aβ (monomers) in aqueous solution at room temperature has previously been stated as approximately 1.7×10^-6^ cm^2^/s (doi:10.1371/journal.pone.0015709). With *h* = 300 nm and *Q*_tot_ = 1.16×10^-16^ m^3^/s this gives *c*_surface_ = 0.33*c*_0_. The diffusivity can be expected to be slightly lower for the oligomers, which from Eq. 2 would give a somewhat higher concentration for the oligomers than the monomers on the surface. The delivery will be localised to the area beneath the pipette and the concentration will drop with distance away from this area. One can show that the concentration will have dropped to approximately half its maximum value (at the position on the surface closest to the pipette), when moving 500 nm along the surface away from the spot right under the pipette[^1^](#_ENREF_1) .

**Supplementary movie 1**

This is a representative 10-minute duration movie of nanopipette dosing of an astrocyte with 3 pM Aβ_42_ oligomers, taken at 1 frame per second.

**Supplementary movie 2**

Movie of Aβ_42_ oligomers on a lipid bilayer imaged using TIRF microscopy at video rates.

**References**

1. Babakinejad, B. et al. Local delivery of molecules from a nanopipette for quantitative receptor mapping on live cells. . *Anal Chem* **85(19)**, 9333-9342. (2013).

2. Picou, R. A. et al. Analysis of Abeta (1-40) and Abeta (1-42) monomer and fibrils by capillary electrophoresis. *Journal of chromatography B, Analytical technologies in the biomedical and life sciences* **879**, 627-632 (2011).
